# Presence of vitamin B_12_ metabolism in the last common ancestor of land plants

**DOI:** 10.1098/rstb.2023.0354

**Published:** 2024-11-18

**Authors:** Richard G. Dorrell, Charlotte Nef, Setsen Altan-Ochir, Chris Bowler, Alison G. Smith

**Affiliations:** ^1^ CNRS, IBPS, Laboratoire de Biologie Computationnelle et Quantitative—UMR 7238, Sorbonne Université, 4 place Jussieu, 75005 Paris, France; ^2^ Institut de Biologie de l’ENS (IBENS), Département de Biologie, École Normale Supérieure, CNRS,INSERM, Université PSL, Paris 75005, France; ^3^ Department of Plant Sciences, University of Cambridge, Cambridge CB2 3EA, UK

**Keywords:** bryophytes, Anthocerotophyta, Marchantiophyta, OneKp, *Chlamydomonas*, phylogenomics

## Abstract

Vitamin B_12_, also known as cobalamin, is an essential organic cofactor for methionine synthase (METH), and is only synthesized by a subset of bacteria. Plants and fungi have an alternative methionine synthase (METE) that does not need B_12_ and are typically considered not to utilize it. Some algae facultatively utilize B_12_ because they encode both METE and METH, while other algae are dependent on B_12_ as they encode METH only. We performed phylogenomic analyses of METE, METH and 11 further proteins involved in B_12_ metabolism across more than 1600 plant and algal genomes and transcriptomes (e.g. from OneKp), demonstrating the presence of B_12_-associated metabolism deep into the streptophytes. METH and five further accessory proteins (MTRR, CblB, CblC, CblD and CblJ) were detected in the hornworts (Anthocerotophyta), and two (CblB and CblJ) were identified in liverworts (Marchantiophyta) in the bryophytes, suggesting a retention of B_12_-metabolism in the last common land plant ancestor. Our data further show more limited distributions for other B_12_-related proteins (MCM and RNR-II) and B_12_ dependency in several algal orders. Finally, considering the collection sites of algae that have lost B_12_ metabolism, we propose freshwater-to-land transitions and symbiotic associations to have been constraining factors for B_12_ availability in early plant evolution.

This article is part of the theme issue ‘The evolution of plant metabolism’.

## 1. Introduction

Since their endosymbiotic origin over a billion years ago, photosynthetic eukaryotes have diversified into a wide range of different lineages with diverse and complex metabolic functions (reviewed in [1]). The majority of photosynthetic eukaryote taxonomic diversity relates to marine and freshwater algae, including unicellular, colonial and multicellular (e.g. giant kelp) [2] forms. Plants only form one very small component, in the subgroup Streptophyta of the broader lineage Viridiplantae (otherwise including Chlorophyta or green algae) [3]. Plants, however, comprise surprising morphological and functional diversity, with two major groups, Tracheophyta (or vascular plants), in which the sporophyte (diploid) form constitutes the majority of the lifecycle, and Bryophyta (including mosses, liverworts and hornworts) in which the gametophyte (haploid) form is predominant [3]. Among these different plant orders, hornworts (or Anthocerotophyta) stand apart through the retention of many features reminiscent of their closest algal relatives. These features include algal-style carbon-concentrating mechanisms (CCM), using a biophysical CCM and pyrenoids [4,5]; an absence of genomic features such as expanded transcriptome factor repertoires and whole-genome duplications associated with vascular plants [6]; and the pervasive adoption of symbioses with cyanobacteria, particularly in the context of nitrogen fixation [7]. As hornworts are projected to have diverged from other plant orders over 400 million years ago, understanding what features link their biology both to vascular plants, and to extant algal relatives in the Streptophyta, may provide us with clues about the deep evolution and terrestrial origin of the land plant lineage [1,6].

One aspect of metabolism that differs between plants and microalgae is the use of vitamin B_12_ (also known as cobalamin) as an enzyme cofactor. B-vitamins are organic micronutrients taken up by organisms from the environment that are essential for central metabolic processes [8]. Plants, fungi and microorganisms also require these compounds for their metabolism but are able to synthesize them *de novo*. The exception is for vitamin B_12_, a cobalt-containing corrinoid molecule that is not synthesized by any eukaryote [9]. In humans, B_12_ is essential for two enzymatic activities: B_12_-dependent methionine synthase (METH) in the C1 cycle [10] and methylmalonyl-CoA mutase (MCM), responsible for the metabolism of odd-chain fatty acids and branched-chain amino acids via the propionate shunt [11]. B_12_ deficiency and its associated pathologies (including pernicious anaemia and methylmalonic acidemia) may be chronic in subsistence economies, with particularly adverse impacts on child development and during pregnancy [12]. Addressing how to mitigate B_12_ deficiency is therefore a key challenge to sustainably feeding a growing planetary population [13,14].

Historically, B_12_ in the human diet has been obtained from different sources. These include from ruminant animals and their derivatives (i.e. dairy products) [15], direct consumption from soil (e.g. via geophagia [16]), from fortified food (nutritional yeast) and from edible seaweeds across the algal tree of life [17]. Indeed, many microalgae also encode METH and/or MCM, and many are B_12_-dependent, with one study finding 171 of 326 algal species were unable to grow in the absence of supplemented B_12_ [18]. Some microalgal lineages may encode further B_12_-dependent enzymes that are not found in humans, such as form II ribonucleotide reductase (RNR-II), first documented in the green Discoba *Euglena* [19].

B_12_ biosynthesis involves over 20 enzymatic steps from the tetrapyrrole intermediate uroporphyrinogen III and has only been described in a subset of bacteria and archaea [20]. Algae may acquire synthesised B_12_ by scavenging from the environment [21], phagotrophic consumption of B_12_-containing organisms [22] or via symbiotic exchanges with B_12_-producing commensals [18]. Additionally, there are a number of B_12_ variants with different axial ligands and different bio-availabilities. Intrinsic factor, the B_12_-binding protein found in the human ileum, has a much higher affinity for cobalamin, which has 5,6-dimethylbenzimidazole (DMB) as the lower axial ligand, than for pseudocobalamin, which has adenine as the lower axial ligand [23]. Eukaryotic microalgae have a similar preference for cobalamin over pseudocobalamin, although a B_12_ remodelling pathway has been documented in some species [24], which enables them to convert pseudocobalamin to cobalamin if supplied with DMB.

**Figure 1.**
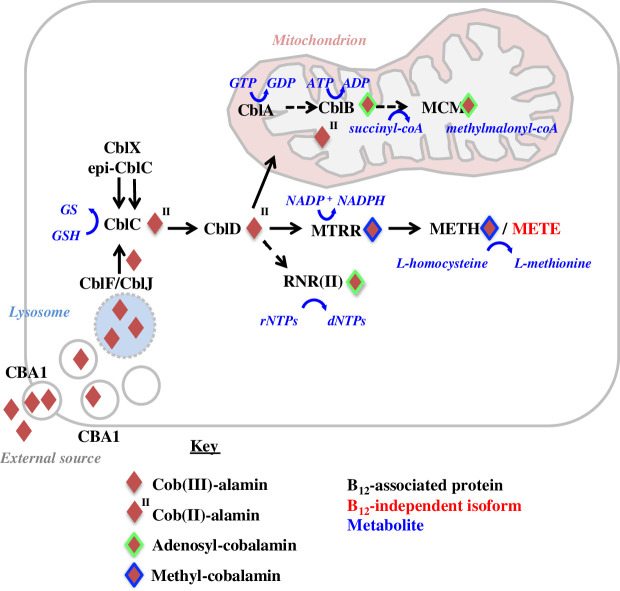
B_12_ uptake and utilization pathways in eukaryotes. A schematic eukaryotic cell is shown with potential B_12_-associated proteins detected in previous studies across the eukaryotes, per [25] and [26]. NB: the roles of the majority of these proteins have been established from human cellular models, and further functional diversity in B_12_ metabolism may exist in non-model species.

Little is known of algal B_12_ uptake or cellular transport, but the pathways are well documented in humans (figure 1). Following its endocytic internalization, cobalamin is released into the cytosol via a chaperone CblC and the lysosomal transporters CblF/CblJ. This involves the reductive removal of any preceding upper-axial ligands associated with the cob(III)-alamin and conversion into cob(I)- and cob(II)-alamins complexed with CblC. Cob(I,II)-alamins complexed with CblC can then be transferred to the conjugate protein CblD, which subsequently directs the assembly of B_12_ into different cobalamin-dependent enzymes [25]. METH requires methylcobalamin, produced in the cytosol from CblD-associated B_12_ by methionine synthase reductase (MTRR) [27], whereas MCM requires adenosylcobalamin, produced in the mitochondria via a probable B_12_ transporter protein CblA [28] and an adenosyltransferase CblB [19]. A few additional proteins (e.g. CblX, epi-CblC) have been shown to have epistatic effects on cobalamin uptake in humans but are of unknown function [25]. Finally, RNR-II is cytosolic and utilizes adenosylcobalamin.

B_12_ may be scarce in the environment, including in soil [29] and large regions of the ocean [30], and uptake and processing of the vitamin are energetically costly [31]. In contrast to the widespread utilization of B_12_ by aquatic algae, land plants have widely lost their metabolic dependence on B_12_ [26]. Plants are able to nonetheless synthesize methionine and complete the methylation cycle in C1 metabolism, despite the loss of METH, because of a B_12_-independent methionine synthase (METE) that arose separately, which can replace METH, albeit with a lower catalytic activity [32]. METE is also present in many METH-containing eukaryotic algae, which renders them facultative B_12_ users [26]. The most parsimonious explanation is an ancestral presence of both METE and METH in eukaryotes with obligately B_12_-dependent species having lost METE, and plants, fungi, and other species that do not have B_12_-associated metabolism having lost METH.

In the last 5 years, the dramatic expansion in plant and algal sequence resources, such as through the OneKp project [3], a global initiative to sequence 1293 publically available transcriptomes from across the plants, and close (particularly freshwater) algal relatives in the Viridiplantae [33], has provided unprecedented insights into early plant evolution. This dataset has offered particular appreciation into the phylogenetic relationships of plants, including the probable monophyly of bryophytes (mosses, liverworts and hornworts) within the plants [3]. Data from within OneKp further support the stepwise accumulation of evolutionary innovations associated with the colonization of land in the closest streptophyte relatives of land plants [34], and the importance of gene losses as well as gene family expansions for the post-terrestrial diversification of both bryophytes and vascular plants [35]. These expanded genomic resources prompted us to revise our current understanding of the distribution of B_12_-dependent metabolism across photosynthetic eukaryotes, considering in which taxonomic and ecological contexts B_12_-dependent metabolism occurs.

## 2. Results

### (a) Distribution of encoded METH/METE proteins indicates B_12_ presence in hornworts

We identified homologues of 13 B_12_-associated proteins shown in figure 1 using available information from algal (*Chlamydomonas reinhardtii*, *Euglena gracilis*, *Phaeodactylum tricornutum*) and human genome sequences (electronic supplementary material, dataset S1, sheet 1). These were used to search a composite genome and transcriptome library of photosynthetic eukaryotes (electronic supplementary material, dataset S1, sheet 2). The library contained: 53 algal and plant genomes, including those of the hornworts *Anthoceros agrestis* and *A. punctigera* [6]; decontaminated versions of the 1000 plant transcriptomes (OneKp; 1293 libraries) [3,33] and the Marine Microbial Eukaryote Transcriptome Sequencing Project (MMETSP; 305 libraries) [36]; and 32 further eukaryotic transcriptomes, as described previously [37]. Certain libraries were represented in more than one library type (e.g. genome plus MMETSP; genome plus OneKp) with 1666 unique strains in the final dataset. Homologues were retrieved by reciprocal BLAST best-hit (RbH), and further evaluated by single-gene RAxML trees, and PFAM domain analysis, and were categorized taxonomically following recently published multi-gene phylogenies of plant and algal diversity [3,37]. Full outputs are provided in electronic supplementary material, dataset S1, sheets 3–4.

**Figure 2.**
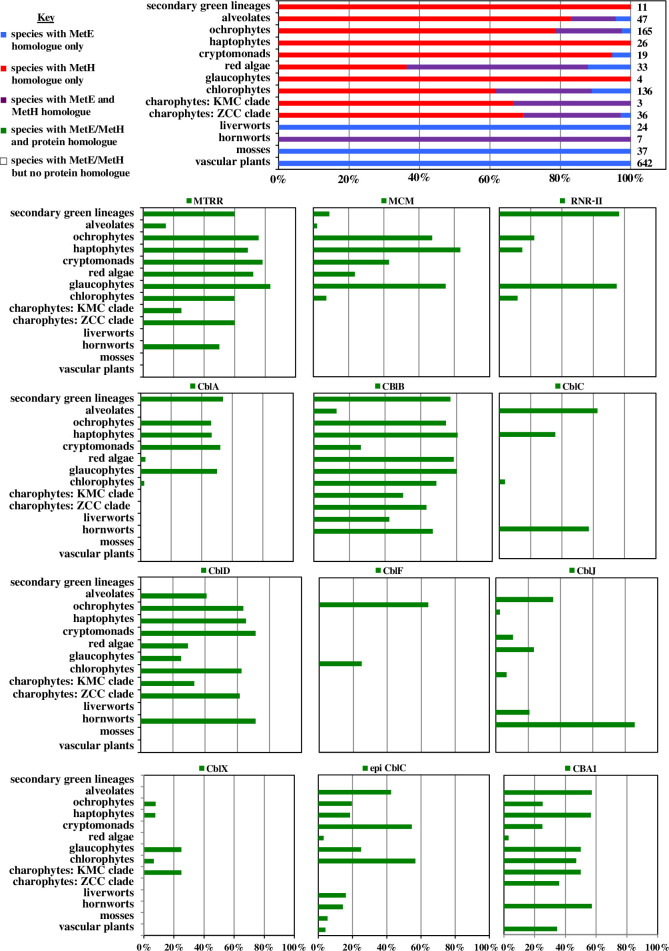
Bar plots of the occurrence of B_12_-associated metabolism across photosynthetic eukaryotes. These graphs show the number of species from 14 different algal phylogenetic or functional groups inferred to possess METH, METE or both, by RbH, PFAM domain and single-gene phylogenetic analysis; and the number of species from these groups for which at least one B_12_-associated enzyme was detected inferred to possess homologues of MTRR, MCM, RNR-II, CBLA, CBLB, CBLC, CBLD, CBLF, CBLJ, CBLX, epi-CBLC or CBA1 via the same methodology. The total number of species assessable in each case is given to the right-hand side of each plot. Complete tabulated occurrences per lineage and species are provided in electronic supplementary material, dataset S1**,** sheets 3–4; and individual homologue lists per gene in electronic supplementary material, dataset S1, sheets 5–18.

The evolutionary distributions of each protein are shown schematically in figure 2. The patterns underline the widespread occurrence of the B_12_-dependent form of methionine synthase, METH, across eukaryotic algae, found in all major marine groups, and in streptophyte relatives of land plants [3]. METH, alongside METE, was further detected in multiple distantly related hornwort genera, suggesting widespread conservation of B_12_-dependent methionine synthases across the class Anthocerotophyta (**e**lectronic supplementary material, dataset S1, sheet 5) [3,5]. These included OneKp homologues from *Megaceros* (*M. tosanus*, OneKp transcript, UCRN2004435; *M. vincentianus*, TCBC2004163), *Nothoceros* (*N. aenigmaticus,* DXOU2038410), *Paraphymatoceros* (*Paraphymato. hallii*, FAJB2057847) and *Phaeoceros* (*Phaeo. carolinianus*, RXRQ2022853, RXRQ2022854), as well as probable METH in *Anthoceros agrestis* Bonn (geneID: Sc2ySwM_228_5027); *A. agrestis* Oxford (utg000003l_252) and *A. punctigera* (utg000098l_165) genomes. METH was not found elsewhere within the land plant lineage, except for one potential METH homologue identified by RbH in the liverwort *Blasia* sp. that resolved during preliminary phylogenetic analyses with bacterial sequences and showed (>99%) high sequence similarity with a *Mucilaginibacter* METH [38]. Previous studies have documented bacterial contamination in the *Blasia* transcriptome [33], and we consider that this is unlikely to be a liverwort METH.

In contrast, the METH phylogeny robustly placed the hornwort sequences with other streptophyte sequences (RAxML bootstrap support: 100%) and as a sister group to the charophytes/coleochaetales (electronic supplementary material, figure S1; electronic supplementary material, dataset S1). This group was positioned more deeply within Viridiplantae, as a sister to the chlorophytes (RAxML bootstrap support: 100%; electronic supplementary material, figure S1). Each of the *Megaceros*, *Nothoceros*, *Phaeoceros* and *Paraphymatoceros* sequences possessed all five PFAMs associated with METH (homocysteine S-methyltransferase, PF02574; pterin-binding, PF00809; B_12_-binding, PF02607 and PF02310; and the activation domain, PF02965) with similar e-values to functionally characterized equivalents from algae (electronic supplementary material, figure S2) [39]. The three identified *Anthoceros* homologues (*A. agrestis* Bonn, 228_5027_1_ *A. agrestis* Oxford utg000003l_252_1; *A. punctigera*, _utg000098l_165_1) possessed all PFAMs apart from the first B_12_-binding (PF02607) domain. However, this PFAM is detected in alternative gene models in each genome (*A. agrestis* Bonn, 362_443; *A. agrestis* Oxford, utg000003l_664; *A. punctigera*, utg000145l_284). We note that at the very least the *A. agrestis* Oxford N- and C-terminal models are encoded on the same chromosomal element (utg000003l), suggesting that *Anthoceros* also possesses an active METH. The hornwort sequences all had conserved active sites (e.g. the substrate-binding pocket of the pterin-binding site) associated with METH activity (electronic supplementary material, figure S2) [39].

We also considered the distribution of the methionine synthase reductase (MTRR) necessary for METH activity. Well-conserved homologues were detected across hornwort genomes and transcriptomes but were absent from other plant groups (figure 2). The hornwort MTRR sequences grouped with other streptophyte sequences within the Viridiplantae (electronic supplementary material, figure S1) and had well-conserved PFAM domains (flavodoxin 1, PF00258 ; FAD-binding-PF00667; oxidoreductase, PF00175; electronic supplementary material, figure S2).

### (b) Widespread occurrence of two distinct METE isoforms in plants

All plant libraries studied possessed *METE* sequences, including hornworts, indicating that both this and *METH* were present in the last common plant ancestor (figure 2). The hornwort *METE* resolved with other plant homologues (electronic supplementary material, figure S1) and possessed well-conserved N-terminal (PF08267) and C-terminal catalytic (PF01717) PFAM domains (electronic supplementary material, figure S2), indicating a vertical origin and possible functionality.

The plant METE enzymes resolved phylogenetically in two discrete families: a conventional isoform with 1768 recovered examples (labelled ‘Clade 1’) and a less frequently observed isoform (‘Clade II’) with 125 observed examples (electronic supplementary material, figure S1; electronic supplementary material, dataset S1). Both isoforms contained both PFAM domains, i.e. are likely to function as methionine synthases (electronic supplementary material, figure S2; electronic supplementary material, dataset S1). In certain cases, Clades I and II were found in the same organism (e.g. the hornworts *Megaceros vincentianus* and *Phaeoceros carolinianus*). Both Clade I and II isoforms were detected in vascular plant, moss, liverwort, hornwort and streptophyte sequences (electronic supplementary material, figure S1), suggesting their presence in the last plant common ancestor. All three characterized *Arabidopsis* methionine synthases (At5g17920; At3g03780; and At5g20980) resolved within Clade 1, but the Clade II enzymes all showed greater proximity by BLASTp analysis to these than to *Arabidopsis* adenosyl-methionine synthases (At1g02500; At4g01850; At3g17390; and At2g36880) (electronic supplementary material, dataset S1, sheet 6) [40]. Both Clade I and II isoforms were distantly positioned to chlorophyte METE sequences, which formed a sister group to red algae, cryptomonads and chromerids (electronic supplementary material, figure S1). The most parsimonious explanation for this distribution would be the ancestral replacement of the streptophyte METE, still retained in chlorophytes, with the Clade I and II isoforms, preceding the loss of METH within specific plant groups.

### (c) Distribution of METE sequences confirms widespread B_12_ auxotrophy in algae

METE was absent from many of the algal libraries searched (figure 2). Transcriptome libraries may under-report the presence of METE, as it may be transcriptionally repressed in B_12_-supplemented cultures [32], and therefore, we limit our consideration to lineages with at least one sequenced genome. Consistent with previous studies [41], the METH and METE distributions indicate B_12_ auxotrophy in haptophytes (including three genomes included: *Emiliania huxleyi*, *Chrysochromulina tobinii* and *Pavlovales* sp. CCMP2436). Only one potential haptophyte METE homologue (from *Pavlova lutheri* UTEX LB1293) was found by RbH but was excluded as a probable green algal contaminant (electronic supplementary material, dataset S1, sheet 21). We note recently discovered and alternative METE-related proteins in the haptophyte *Phaeocystis*, discussed below [42]. No occurrences of METE were found in the prasinophyte class Mamiellophyceae (including *Micromonas* and *Ostreococcus* sp. genomes) or the ochrophyte class Pelagophyceae (including two genomes: *Aureococcus anophagefferens* and Pelagophyceae sp. CCMP2097) (figure 2). The absence of METE from these three groups is particularly interesting given their environmental abundance [43], underlining the importance of B_12_ acquisition for marine photosynthesis.

Further instances of obligate B_12_ requirements were found at more phylogenetically localized scales, including the most immediate relatives of the model green algal species *C. reinhardtii* [44] and the glaucophytes, considered as the sister group to the Viridiplantae; and Palmophyllophyceae, either a sister group to all other chlorophytes or to all other Viridiplantae [45,46]. Finally, dinoflagellates (including three genomes from *Symbiodinium* sp.) were found to possess only partial METE sequences, which lacked the conventional N-terminal PFAM domain (MetH_synt_1; PF08267), following previous studies [39] (electronic supplementary material, dataset S1). We did, however, identify complete METE sequences containing both PFAM domains in the chromerids *Chromera velia* and *Vitrella brassicaformis*, which are the closest photosynthetic relatives of dinoflagellates [47]. The dinoflagellate METE-like sequences were distantly related to other algae and instead resolved with Archaea (electronic supplementary material, figure S1), which likewise lacked the N-terminal PFAM domain. This may suggest a horizontal acquisition of the dinoflagellate METE-like gene accompanied by the loss of the complete METE isoform.

Finally, the METE sequences of the red algae *Porphyridium* and *Cyanidioschyzon* and the chrysophyte *Poteriospumella* resolved with bacterial homologues (electronic supplementary material, dataset S1; electronic supplementary material, figure S1). In each case, these acquisitions were shared between multiple related strains and are likely to be bacterial horizontal gene transfers as opposed to contaminants.

### (d) MCM and RNR-II enzymes are restricted to individual algal lineages

Next, we considered the distributions of B_12_-dependent MCM and RNR-II across our dataset (figure 2). Neither enzyme was detected in streptophytes (including hornworts) and was absent from chlorophytes, except for sporadic occurrences that are likely consistent with horizontal acquisitions (discussed below). MCM homologues were primarily detected in algae with secondary red chloroplasts (e.g. diatoms, cryptomonads, haptophytes, ochrophytes), typically possessing well-conserved PFAM domains, and predictable mitochondrial-targeting sequences (electronic supplementary material, dataset S1; electronic supplementary material, figure S3). MCM is implicated in the catabolism of branched-chain amino acids in diatoms under nitrate exhaustion but lacks an apparent RNAi mutant phenotype [48], so its significance for B_12_ dependence in eukaryotic algae remains to be determined. We additionally detected MCM in a small number of glaucophytes, red algae (*Galdieria sulphuraria*) and green algae (*Tetraselmis*, *Chlorella*) (figure 2). The MCM phylogeny retrieves a sister group relationship between green algae and haptophytes (electronic supplementary material, figure S1), implying it was probably acquitted by horizontal gene transfer.

**Figure 3.**
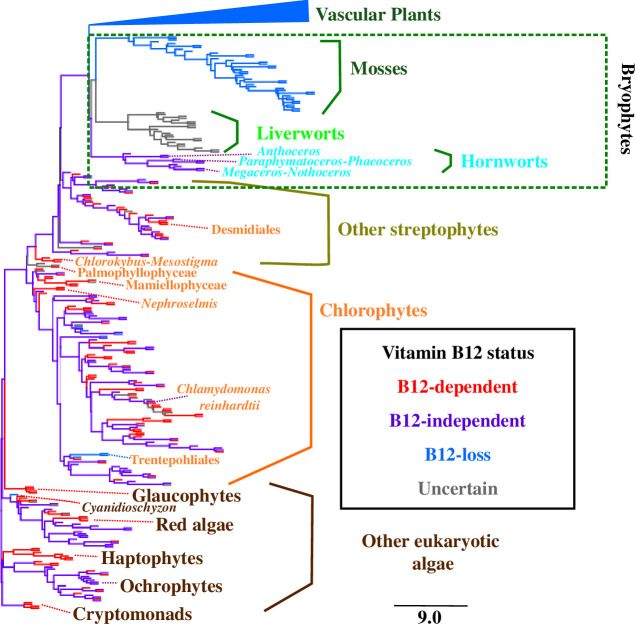
Vertical inheritance of hornwort B_12_ metabolism. This figure shows a concatenated multi-gene tree topology for all OneKp transcriptomes, taken from (3). Taxa names are coloured by origin: dark green, vascular plants and mosses; light green, liverworts; cyan, hornworts; dark yellow, other streptophytes; orange, chlorophytes; brown, other algae. Branches are shaded by B_12_ status: red, B_12_-dependent metabolism only; purple, B_12_-dependent and B_12_-independent metabolism; light blue, B_12_-independent metabolism only; grey, unknown. Vascular plants are collapsed to a single branch. Liverworts are marked as ‘unknown’ due to the uncertain function of their encoded CblB and CblJ proteins. Major algal lineages are labelled, alongside species of interest. A nexus format topology is provided in electronic supplementary material, dataset S1, sheet 22.

The B_12_-dependent type II ribonucleotide reductase (RNR-II), first detected in the secondary green chloroplast-containing alga *Euglena gracilis* [49], was also found in the distantly related haptophytes and chlorarachniophytes, reflecting possible ancestral horizontal gene transfers between all three lineages [50]. Intriguingly, it is also present in the Chlamydomonadales (although not *C. reinhardtii*), where it has most likely been acquired by an independent horizontal gene transfer from bacteria (figures 2 and 3).

### (e) Distribution of uptake proteins suggests deeper retention of B_12_-dependent metabolism within the bryophytes

We further considered the distribution of six proteins associated with the uptake and intracellular transport of B_12_ (CblA, B, C, D, F and J) and two epistatic regulators in humans (CblX and epi-CblC) [25]. We could identify widespread presence of CblC, D and J proteins in hornwort libraries (figure 2). The hornwort sequences resolved within clades of other Viridiplantae and contained well-conserved PFAM domains (electronic supplementary material, figures S2 and S4), suggesting vertical origin and functionality. That said, very few carried credible signal peptides that might suggest participation in an endosomal B_12_ uptake pathway (electronic supplementary material, figure S3). The only protein for which we could identify consistent endomembrane targeting predictions was CblF (electronic supplementary material, figure S3), but this was almost exclusively detected among dinoflagellates (figure 2; electronic supplementary material, figure S4). We tentatively propose that the B_12_ intracellular trafficking pathways may not be identical across different branches of the eukaryotic tree of life.

Surprisingly, homologues of CblB, responsible for the synthesis of adenosylcobalamin [19], were found in hornworts (figure 2; electronic supplementary material, figure S1). This was despite the absence of MCM, which uses adenosylcobalamin, alongside the CblB partner enzyme CblA (figure 2) [28]. We could further detect homologues of CblB and indeed CblJ in liverworts, which grouped monophyletically with homologues from hornworts (electronic supplementary material, figure S4) and possessed well-conserved PFAM domains (electronic supplementary material, figure S2). It remains to be determined (e.g. via the functional characterization of putative adenosylcobalamin-dependent enzymes) if some form of adenosylcobalamin-dependent metabolism is retained in hornworts and liverworts.

We also searched for homologues of the newly characterized CBA1 protein that is required for cellular B_12_ uptake in the green alga *C. reinhardtii* and the diatom *P. tricornutum* [51]*,* using similar methodology. While the *Chlamydomonas* and *Phaedoactylum* CBA1 proteins do not possess the same PFAM domains, they show moderate reciprocal similarity to one another (BLASTp pairwise identity of 27.8%; e-value 1 × 10^−14^) and thus were used to construct a consensus hmm for validation of individual sequences. Consistent with the findings of the initial study documenting its role [51], potential CBA1 homologues were found across the tree of life, including in hornworts (figure 2; electronic supplementary material, figure S4), grouping within a monophyletic clade of streptophyte sequences that suggest vertical inheritance. Many of the CBA1 homologues possessed signal peptides and could thus have potential extracellular or plasma membrane localizations (electronic supplementary material, figure S3). That said, plausible CBA1 homologues were found using the same approach in vascular plants, although not in mosses or liverworts. Thus, we tentatively propose that hornworts could uptake extracellular B_12_ using CBA1-type proteins, although the role of CBA1 homologues in hornworts and indeed in other plant species awaits experimental validation, e.g. via complementation assays with *Chlamydomonas* CBA1 mutants [51].

### (f) Rare occurrences of algae lacking known B_12_-dependent metabolism are biased towards terrestrial and symbiotic species

Finally, we explored which algae in our dataset, like vascular plants, may have dispensed with known B_12_-associated metabolism. Within our dataset, 11 of the algal libraries were found to possess METE but lacked identifiable METH/MTRR, RNR-II or MCM activities (table 1). We further identified four species that possess METE and either MCM or RNR-II but lack METH/MTRR, suggesting a loss of B_12_-related methionine synthesis (table 1).

**Table 1.** Algal species lacking identifiable B_12_-associated metabolism, based on the retention of METE and the absence of METH and MTRR sequences. A map of collection sites of all algae included in the dataset is provided at https://www.google.com/maps/d/viewer?hl=en&mid=1wpY67NIYonDugMXiA1g7wdcET9Qd3CU&ll.

species	lineage	strain	ecological context	collection latitude	collection longitude	notes
*Blastophysa cf. rhizopus*	chlorophytes	M3368	marine, epiphyte	28.4	14.4	based on PFAM only
*Botryococcus braunii*	chlorophytes	UTEX 2441	freshwater	−13.5	−72.1	based on PFAM only
*Brandtodinium nutriculum*	dinoflagellates	RCC3387	marine, foraminiferan symbiont	43.7	7.3	
*Cephaleuros virescens*	chlorophytes	SAG 28.93	leaf parasite	−29.9	31	based on PFAM only; Trentepohliales
*Coccomyxa pringsheimii*	chlorophytes	SAG 216.7	freshwater, lichen symbiont	N/A	N/A	
*Cyanidioschyzon merolae*	red algae	10D	hot spring	40.9	14.3	
*Galdieria sulphuraria*	red algae	074W	fumarole	−7.6	11.2	encodes MCM, CblA
*Geminigera cryophila*	cryptomonads	CCMP2564	marine, Antarctic	−77.8	−163	
*Ignatius tetrasporus*	chlorophytes	UTEX2012	freshwater	N/A	N/A	may encode RNR-II
*Leptosira obovata*	chlorophytes	SAG 445.1	freshwater	47.5	7.9	
*Porphyridium cruentum*	red algae	UTEX 161	freshwater	47.6	7.6	encodes CblB
*Poteriospumella* JBC07	chrysophytes	JBC07	freshwater	31.5	120.2	
*Pseudoscourfieldia marina*	chlorophytes	SCCAP K/0017	estuarine	59.4	10.6	encodes CblB
*Trentepohlia annulata*	chlorophytes	SAG 20.94	freshwater	49.7	16	based on PFAM only; Trentepohliales
*Vitrella brassicaformis*	alveolates	CCMP3155	marine, coral	−23.5	152	

The species that lack identifiable B_12_-dependent metabolism mainly originate from freshwater (e.g. *Leptosira obovata*, *Porphyridium cruentum*, *Poteriospumella* JBC07) or terrestrial habitats (*Coccomyxa pringsheimii*, *Cyanidioschyzon merolae*, *Galdieria sulphuraria*). Freshwater habitats may be limited by the abundance of B_12_-producing bacteria in the water column (as opposed to sediments) [52] and in certain cases by cobalt scarcity [53]. Similarly, *Geminigera cryophila*, which is the only cryptomonad within our dataset to lack METH and encode METE, was collected from the Antarctic, which is characterized by vitamin B_12_ limitation (figure 2) [30]. Finally, many of the algal species that lack B_12_-dependent metabolism are symbionts of other organisms (*Leptosira obovata* and Trentepohliales, from lichens [54]; *V. brassicaformis*, from coral [47]; and *Brandtodinium nutriculum*, a foraminiferan symbiont [55]) and may be subject to B_12_ limitation, or even potentially receive methionine and as folate from their hosts.

## 3. Discussion

We have queried the distribution of 13 gene families encoded B_12_-associated proteins across more than 1600 plant and algal genomes and transcriptomes, in particular profiting from sequence data from the OneKp transcriptome project [3] (figure 1, electronic supplementary material, dataset S1, sheet 2). Our data suggest the hitherto undocumented retention of vertically inherited B_12_-associated methionine synthesis pathways (METH and MTRR) and B_12_ uptake-associated proteins (CblB, C, D and J) in hornworts and the retention of potential CblB and CblJ homologues in liverworts (figure 2). Formally, the active use of B_12_ by hornworts awaits functional characterization, such as by bioassay of B_12_ content [56], from experimental inference of B_12_ use such as B_12_-dependent suppression of hornwort METE [32] or via uptake of fluorescently labelled B_12_ analogues [57] by hornwort cells.

Overall, our data indicate the presence of B_12_-dependent metabolism in the last common plant ancestor following the transition to land, with subsequent losses in mosses, vascular plants and (dependent on the functions of the detected CblB and CblJ homologues) liverworts (figure 3). It remains to be determined when this loss occurred during plant evolution. Some studies have proposed hornworts as the earliest-diverging land plant group [58,59], which would imply a single METH loss in the common ancestor of liverworts, mosses and vascular plants. Other studies, including the *Anthoceros* genome and OneKp transcriptome projects [3,6,35], strongly recover bryophyte monophyly, indicating independent losses of B_12_-dependent metabolism in vascular plants and mosses (figure 3). In either case, the presence of B_12_-dependent methionine synthesis in hornworts sits alongside other phenomena (e.g. biophysical CCMs in hornworts [5] and auxin transporters in charophytes [34]) that blur the physiological boundaries between algae and plants.

It remains to be determined why B_12_-dependent metabolism was lost in early plant lineages. Many hornwort species are characterized by the presence of cyanobacterial symbionts, which might produce pseudocobalamin, although this is not bioavailable to microalgal species [23,60]. It is possible that hornworts can utilize pseudocobalamin, and thus, the retention of METH may be linked to their microbiome. Equally, the repeated losses of METH and its associated enzymes in other freshwater, terrestrial and symbiotic algae may suggest specific ecological niches in which the loss of B_12_-associated metabolism is more likely to have occurred (table 1). Comparative studies of the cell biology of these species may elucidate reasons for the loss of B_12_ metabolism in most land plant lineages.

The data presented in our current study are based on B_12_-associated proteins with known structures and functions from model species and relate only to the diversity of these proteins across plants and algae. Beyond these known pathways and functions, yet undetected diversity may exist in B_12_ metabolism across the tree of life. A recent study identified a novel fusion gene product that appears to confer B_12_-independent methionine synthesis in haptophytes, projected in our data and elsewhere to have universally lost METE [41,42] (figure 2). Deeper inspection of plant and algal genomes and meta-genome assembled genomes (e.g. from the *Tara* Oceans expedition) for novel genes showing structural similarity to the full diversity of archaeal, bacterial and eukaryotic B_12_-independent synthases, may provide clues about the diversity of strategies used by plants and algae for tolerating environmental B_12_ scarcity [61,62].

Ultimately, determining why most plants, excluding hornworts, have apparently lost B_12_-associated metabolism is particularly important due to the prevalence of vitamin B_12_ insufficiency/deficiency in populations consuming plant-based diets [12]. Understanding the significance of B_12_ utilization across algae may facilitate the reintroduction of B_12_ uptake into crop species, or algal cultivation for dietary consumption [57], sustainably feeding the human planetary population [14].

## 4. Material and methods

### (a) Homologue detection

Potential homologues of 13 vitamin B_12_-associated enzymes (listed in electronic supplementary material, dataset S1, sheet 1) [26] were searched across a composite library of 1663 non-redundant plant and algal genomes and transcriptomes (electronic supplementary material, dataset S1, sheets 2–4) [3,36,37] by BLASTp with threshold e-value 10^−05^. Potential matching sequences were extracted and searched by BLASTp against the complete *Arabidopsis thaliana* genome [63], which uniquely encodes METE, supplemented with the query sequences defined previously. Sequences that retrieved a best-scoring match to a query sequence were retained for downstream phylogenetic analysis (electronic supplementary material, dataset S1, sheets 5–17). For METE which is retained in plants, sequences that retrieved one of the three *Arabidopsis* methionine synthases were likewise retained for downstream analysis [40].

### (b) Phylogeny

Inferred homologues were aligned against the query sequence, and best-scoring homologues obtained from parallel BLASTp searches of 51 combined genome and transcriptome libraries corresponding to different prokaryotic and non-photosynthetic eukaryotic taxonomic groups from across the tree of life [37] by MAFFT v. 7.487 using the --auto setting [64]. The resulting alignments were imported into GeneIOUS v. 10.0.9 and initially screened using the in-built NJ tree function with 100 replicates and random starting seeds and highly divergent branches (defined visually as branches with >1.0 calculated substitutions per site) were iteratively removed [65]. Curated alignments were trimmed with trimal v. 1.4 using the --gt 0.5 setting [66] and then inspected with RAxML v. 8.0 using the PROTGAMMAJTT substitution matrix and 350 bootstrap replicates [67]. The best-scoring tree was inspected for branches of contaminant origin (e.g. sequences from one algal library only that resolved with bacterial homologues). Homologues that passed the initial RAxML curation were passed through a second iteration of mafft alignment, manual curation and RaxML phylogeny, prior to enumeration of homologue presence/absence (electronic supplementary material, dataset S1, sheets 18–21).

### (c) Functional, targeting and biogeographical annotation

PFAM domains were searched in each homologue retrieved by the initial RbH search by HMMER v. 3.3.2 against the Pfam v. 35.0 library [68]. Sequences that retrieved PFAM domains associated with each query protein with threshold e-value 10^−05^ were recorded (electronic supplementary material, dataset S1, sheet 22). For CBA1, where no PFAM is associated with the *C. reinhardtii* sequence, a specific hmm was generated from the characterized *Phaeodactylum* and *Chlamydomonas* proteins and used to screen homologues (electronic supplementary material, dataset S1, sheet 23). Localizations for each protein were inferred following previous studies [37], considering the consensus predictions of WolfPSort, SignalP, ASAFind, TargetP and HECTAR (electronic supplementary material, dataset S1, sheet 23). Collection sites were recorded for each algal species using the corresponding culture collection records considering strain synonyms. Where appropriate, direct information was obtained from the literature or collector [69–71].

## Data Availability

Full supporting data for this project, including retrieved homologues, alignment and tree topologies, are provided in dataset S1. An interactive map of all algal collection sites identified within the dataset, shadeable either via phylogenetic affiliation or inferred vitamin B_12_ metabolic status, is available via https://www.google.com/maps/d/viewer?hl=en&mid=1wpY67NIYonDugMXiA1g7wdcET9Qd3CU&ll. Supplementary material is available online [72].
